# Microsatellite Variation in the Most Devastating Beetle Pests (Coleoptera: Curculionidae) of Agricultural and Forest Crops

**DOI:** 10.3390/ijms23179847

**Published:** 2022-08-30

**Authors:** Manee M. Manee, Badr M. Al-Shomrani, Musaad A. Altammami, Hamadttu A. F. El-Shafie, Atheer A. Alsayah, Fahad M. Alhoshani, Fahad H. Alqahtani

**Affiliations:** 1 National Center for Bioinformatics, King Abdulaziz City for Science and Technology, Riyadh 11442, Saudi Arabia; 2 National Center for Agricultural Technology, King Abdulaziz City for Science and Technology, Riyadh 11442, Saudi Arabia; 3 Department of Life Sciences, Imperial College London, London SW7 2AZ, UK; 4 Date Palm Research Center of Excellence, King Faisal University, Al-Ahsa 31982, Saudi Arabia; 5 National Center for Biotechnology, King Abdulaziz City for Science and Technology, Riyadh 11442, Saudi Arabia

**Keywords:** curculionidae, red palm weevil, microsatellite, genome assembly, SSR abundance

## Abstract

Weevils, classified in the family Curculionidae (true weevils), constitute a group of phytophagous insects of which many species are considered significant pests of crops. Within this family, the red palm weevil (RPW), *Rhynchophorus ferrugineus*, has an integral role in destroying crops and has invaded all countries of the Middle East and many in North Africa, Southern Europe, Southeast Asia, Oceania, and the Caribbean Islands. Simple sequence repeats (SSRs), also termed microsatellites, have become the DNA marker technology most applied to study population structure, evolution, and genetic diversity. Although these markers have been widely examined in many mammalian and plant species, and draft genome assemblies are available for many species of true weevils, very little is yet known about SSRs in weevil genomes. Here we carried out a comparative analysis examining and comparing the relative abundance, relative density, and GC content of SSRs in previously sequenced draft genomes of nine true weevils, with an emphasis on *R. ferrugineus*. We also used Illumina paired-end sequencing to generate draft sequence for adult female RPW and characterized it in terms of perfect SSRs with 1–6 bp nucleotide motifs. Among weevil genomes, mono- to trinucleotide SSRs were the most frequent, and mono-, di-, and hexanucleotide SSRs exhibited the highest GC content. In these draft genomes, SSR number and genome size were significantly correlated. This work will aid our understanding of the genome architecture and evolution of Curculionidae weevils and facilitate exploring SSR molecular marker development in these species.

## 1. Introduction

The family Curculionidae represents a highly diverse group of coleopteran insects that differ morphologically, ecologically, and behaviorally. Specifically, it comprises 17 subfamilies with over 50,000 described species [1,2]. Members of this family are generally called weevils (snout beetles), and most have a characteristic snout or beak, which is an elongation of the forepart of the head. Curculionidae includes the most damaging and devastating pests of horticultural, field, and forest crops in various ecosystems including rainforests, deserts, and grasslands; these species pose a real menace to global agricultural and forest produce [3,4,5]. For example, the rice weevil, *Sitophilus oryzae*, can cause 10–80% yield loss [6]. Meanwhile, the mountain pine beetle, *Dendroctonus ponderosae*, is considered the most important mortality agent for forest ecosystems in western North America and Europe. This weevil seriously influences deforestation and global carbon sequestration strategies [7,8]. Similarly, species of the genus *Rhynchophorus*, called palm weevils, cause substantial direct damage to several palms of economic importance, such as the edible date palm, oil palm, coconut palm, and the ornamental Canary Islands date palm [5]. They also damage palms indirectly through vectoring diseases or creating wounds that allow the entry of other pathogens [9,10]. Palm weevils also negatively affect the aesthetic value of palms used in urban landscape design [5].

Weevils also comprise extremely important invasive species that may present quarantine problems if they gain entry into new areas, which in modern times is more likely due to the global commercialization and movement of agricultural and forest products [11]. Moreover, it is not easy to detect these weevils during early stages of infestation, making them extremely difficult to control. Nevertheless, it is possible to manage weevils through combining cultural, biological, and chemical strategies in an integrated pest management program. When setting up such control strategies, proper identification and classification of the target beetles is essential to ensure their appropriateness [3]. Recently, [12] reported the first phylogenetic analysis of the subfamily Dryophthoridae within the family Curculionidae which is essential for proper identification and classification.

Microsatellites, also known as simple sequence repeats (SSRs), are 1–6 bp motifs present in both coding and non-coding regions of eukaryotic and prokaryotic genomes that have become the primary source of genetic markers for population analysis in insects due to their high levels of polymorphism [13]. It is well established that SSRs have high rates of mutation and thus have implications for genome organization and genetic variation [14,15]. In addition, SSRs play essential roles in genetic divergence and phenotypic diversity, aiding species in adapting to different environments [16]. Generation of SSR markers by using conventional methods has been challenging; however, in silico mining and analysis of SSRs has proven an effective approach.

To date, draft genome sequences have been released for nine species in the Curculionidae family: *R. ferrugineus*, *Sitophilus oryzae*, *Hypothenemus hampei*, *D. ponderosae*, *Pissodes strobi*, *Elaeidobius kamerunicus*, *Ips nitidus*, *Listronotus oregonensis*, and *Listronotus bonariensis*. This study aimed to identify and characterize microsatellites in the draft genomes of these major agricultural insect pests. The obtained data may contribute to ongoing efforts in managing this group of weevils.

## 2. Materials and Methods

### 2.1. Collection of Insect Samples

The female adult of the red palm weevil (RPW) *R. ferrugineus* used for this study was randomly selected from a colony reared at the insectary of the Date Palm Research Center of Excellence, King Faisal University, Saudi Arabia. The weevil was sexed based on the absence (female) of tuft hairs on the dorsal side of the rostrum [17]. The initial adult weevils used to start the colony were captured in pheromone-food baited traps deployed in an infested date palm plantation in Al-Ahsa, Saudi Arabia (Latitude: 25.268528${}^{\circ }$ N, Longitude: 49.707218${}^{\circ }$ E). The weevil colony has been kept for at least three generations, feeding on sugar cane and bolts of the popular “Khalas” date palm cultivar.

### 2.2. Sample Preparation and DNA Extraction

Tissue (20–30 mg) was obtained from adult female RPW for DNA extraction. Lysis buffer (600 μL) consisting of 10 mM Tris-HCl, 400 mM NaCl, 100 mM EDTA, pH 8.0, 40 μL 10% SDS, and 10 μL Proteinase K (Qiagen, cat. no. 19131; Hilden, Germany) was added to the tissue and incubated overnight, after which the sample was centrifuged and the supernatant discarded. Pellets were resuspended in 1 mL PBS, then processed for DNA extraction and purification by using the KingFisher^TM^ Flex Purification System (ThermoFisher Scientific, cat. no. 5400610; Waltham, MA, USA) and MagMAX^TM^ DNA Multi-Sample Ultra 2.0 Kit (Applied Biosystems, cat. no. A36570; Waltham, MA, USA). The obtained DNA was quantified by using the Qubit dsDNA BR Assay Kit (Invitrogen, cat. no. Q32850; Waltham, MA, USA).

### 2.3. Next-Generation Sequencing and Genome Assembly

Whole-genome sequencing was outsourced to Macrogen (South Korea) and used paired-end sequencing with read length 151 nucleotides. Library preparation was carried out by using a TruSeq Nano DNA kit according to the sample library preparation protocol (Part # 15041110 Rev. D) on an Illumina NovaSeq 6000 System. De novo assembly was carried out by using SPAdes v3.13.1 with k-mer sizes of 21, 33, 55, and 77 [18]. QUAST v5.2.0 was used to assess the draft assembly metrics [19]. Draft genome completeness was evaluated with the Benchmarking Universal Single-Copy Orthologs (BUSCO) v4.0.6 [20] and the Arthropoda gene set (1013 genes).

### 2.4. Genome Sequences

The draft genome sequences of nine crop pests were selected for analysis of SSR distributions at genome level. These sequences were assembled at scaffold level according to the genomic resources of the NCBI. The genome sequences in FASTA format were obtained from the Genomes FTP site (ftp://ftp.ncbi.nlm.nih.gov/genomes/ (accessed on 16 May 2022)) and had the following accession numbers: GCA_012979105.1 (male RPW), GCA_014462685.1 (RPW larva), GCA_002938485.2 (*S. oryzae*), GCA_013372445.1 (*H. hampei*), GCA_020466585.1 (female *D. ponderosae*), GCA_020466635.1 (male *D. ponderosae*), GCA_016904865.1 (*P. strobi*), GCA_014849505.1 (*E. kamerunicus*), GCA_018691245.1 (*I. nitidus*), GCA_019359885.1 (*L. oregonensis*), and GCA_014170235.1 (*L. bonariensis*). Although unknown at the time of sequencing, the sex of the RPW larva sample was inferred to be female after analysis of male/female coverage ratios.

The completeness of the assemblies was assessed in relation to BUSCO v4.0.6 [20] based on the Arthropoda database (1013 genes). When investigating the distribution of SSRs in different genomic regions, only three draft genomes and corresponding GFF annotation files could be used: the *R. ferrugineus* larva and *D. ponderosae* male and female specimens. We also included the GFF file of *Tribolium castaneum* (red flour beetle, family Tenebrionidae) for comparison purposes.

### 2.5. Identification of Microsatellites

The software PERF v0.2.5 [21] was used to scan each entire genome and conduct genome-wide SSR mining. The following criteria were adopted to identify perfect SSRs: repeat lengths of 1 to 6 nucleotides and minimum repeat numbers of 12 repeats for mononucleotides, 7 repeats for dinucleotides, 5 repeats for trinucleotides, and 4 repeats for tetra-, penta- and hexanucleotides; these criteria are consistent with previous studies [22,23]. The remaining parameters were set as default. Repeats with unit patterns being circular permutations and/or reverse complements were deemed as a single type in this study [24,25]; for instance, depending on the reading frame and strand, the type “ACT” corresponds to ACT, CTA, TAC, ATG, GAT, and TGA. The relative frequency (number per Mb) and relative density (length in bp per Mb) of SSRs were utilized in comparing different types of SSR repeats or motifs.

### 2.6. Assigning Microsatellites to Genomic Regions

We determined exon sequences and gene coding sequences (CDSs) of the nine weevil genomes in this study according to the positions noted in genome annotation files in general feature format (GFF). Intergenic regions were defined as the interval sequences between two adjacent genes. Intronic regions were defined as interval sequences within genes that did not overlap any annotated exons. We identified the coordinates defining intergenic and intronic regions from GFF files by using the BEDtools subtract tool v2.30.0, and assigned the identified perfect SSRs to genomic compartments by using the BEDtools intersect tool v2.30.0 [26].

### 2.7. Statistical Analysis

All graphical and statistical analyses were carried out in the R programming environment (v4.0.4) (R Core Team, 2021). Pearson correlations determined by using the cor.test method were utilized to elucidate correlations between SSR data sets, including in terms of the number, relative frequency, relative density, and GC content of SSRs.

## 3. Results

### 3.1. Genome Assembly and Assessing of Draft Genome Completeness

**Figure 1:**
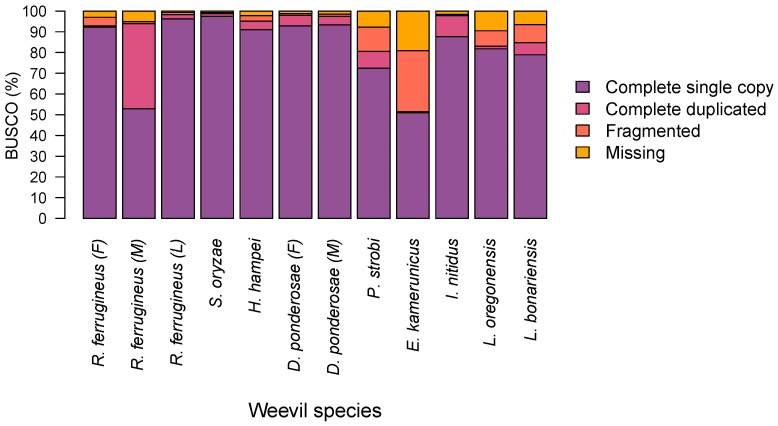
Assessment of the assembled weevil genomes using the arthropoda_odb10 BUSCO dataset (1013 single-copy orthologs).

The de novo assembly of female RPW was performed, generating a draft genome of 1121.36 Mb with a GC content of 43.96%. Contigs with lengths less than 200 bp were filtered out prior to the analysis. The final draft assembly resulted in 945,214 contigs that yielded the longest contig length of 720,101 bp with an N50 contig length of 7782 bp. To determine the completeness of each weevil genome assembly including our female RPW draft, we compared it against the BUSCO Arthropoda lineage dataset (arthropoda_odb10), which consisted of 1013 single-copy orthologs. This revealed that for eight of the sequenced species, 72.4–97.4% of those 1013 Arthropoda single-copy orthologs were completely present; the exception were *R. ferrugineus* adult male and *E. kamerunicus*, at 52.9% and 51%, respectively (Figure 1).

### 3.2. Identification and Characterization of Microsatellites in Beetle Genomes

**Figure 2:**
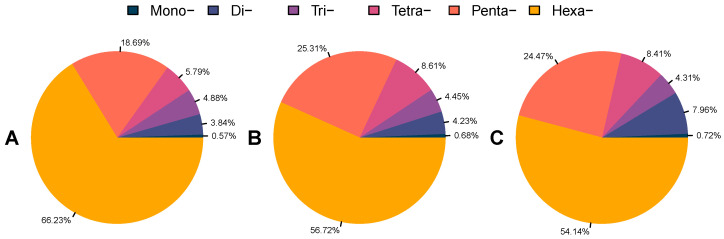
Type composition of SSRs identified in the RPW genome assemblies without applying any search criteria. (**A**–**C**) represent adult female, adult male, and larval samples, respectively.

Twelve draft genomes representing the insect species *R. ferrugineus*, *S. oryzae*, *H. hampei*, *D. ponderosae*, *P. strobi*, *E. kamerunicus*, *I. nitidus*, *L. oregonensis*, and *L. bonariensis* were scanned for perfect microsatellites by using PERF. We first carried out analyses to report all perfect SSRs in the RPW genomes without applying any search criteria (Supplementary Files S1–S3). All exhibited similar patterns of SSRs, as shown in Figure 2. When applying consistent search parameters, a total of 57,175, 50,723, and 67,261 perfect SSRs were identified with frequencies ranging from 50.99 to 114.11 SSRs/Mb in the adult female, adult male, and larval RPW genomes, respectively (Table 1). These perfect SSRs occupied about 0.13%, 0.14%, and 0.36% of the respective genome, had mean lengths of 25.91, 22.29, and 31.98 bp, and their relative densities ranged from 1320.92–3649.45 SSRs/Mb. The other true weevil genomes exhibited similar length proportions for their SSRs, ranging from 0.02% (*E. kamerunicus*) to 1.44% (*L. oregonensis*), as seen in Table 1. Number of SSRs was positively correlated with their relative frequency and density (Pearson *r* = 0.944, *p* < 0.01 and Pearson *r* = 0.937, *p* < 0.01, respectively). The genome size of these draft genomes was also significantly positively correlated with number of SSRs (Pearson *r* < 0.580, *p* < 0.05). In contrast, the GC content of SSRs was not significantly correlated with number of SSRs (Pearson *r* < −0.442, *p* = 0.150). The relative frequency and density of SSRs were also not significantly correlated with genome size (Pearson *r* < 0.370, *p* = 0.236 and Pearson *r* < 0.324, *p* = 0.305, respectively). For example, *P. strobi* has the largest genome (2025.02 Mb) among those surveyed, but was found to have lower SSR frequency (76.30 SSRs/Mb) compared to some other species with smaller genome sizes (Table 1).

**Figure 3:**
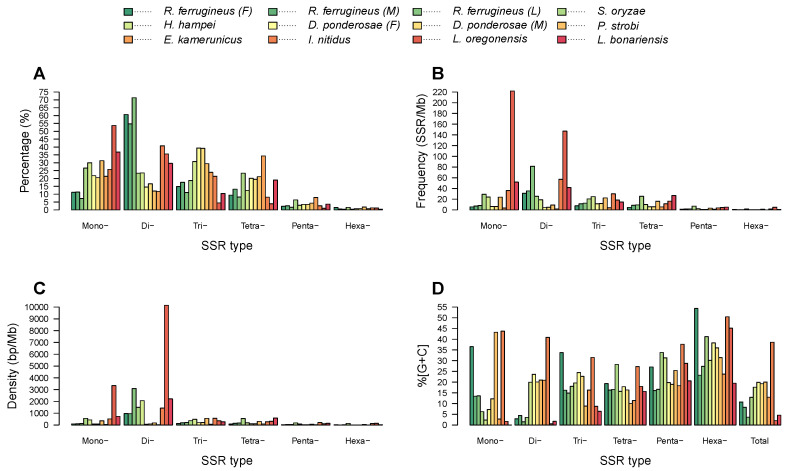
Comparison of SSR types according to their percentage, frequency, density, and GC content in the weevil genome assemblies. Percentages were determined by dividing the number of SSRs of a given type by the total number for that species. (**A**–**D**) represent the percentage, frequency, density, and GC content of SSRs, respectively.

Table 2 lists the respective number, length, relative frequency, relative density, and percentage of each of the six types of SSRs. The percentage and relative frequencies and densities of different SSR types were found to vary in the twelve draft genomes (Figure 3). Dinucleotide SSRs were the most frequent type in the *R. ferrugineus* adult male, adult female, and larva and in *I. nitidus*, with respective frequencies of 30.93, 35.52, 81.43, and 57.18 SSRs/Mb; these accounted for 60.66%, 54.77%, 71.35%, and 40.78% of SSRs in those draft genomes (Figure 3A,B). Meanwhile, mononucleotide SSRs were the most abundant type in *S. oryzae*, *P. strobi*, *L. oregonensis*, and *L. bonariensis*, with respective frequencies of 29.18, 23.87, 221.81, and 51.89 SSRs/Mb and comprising 26.64%, 31.29%, 53.71%, and 36.83% of all SSRs (Figure 3A). Trinucleotide SSRs were the most frequent type in *H. hampei* and in both female and male *D. ponderosae*, with frequencies of 24.78, 11.43, and 11.85 SSRs/Mb. Finally, tetranucleotide SSRs were the most abundant type in *E. kamerunicus*, with a frequency of 5.60 SSRs/Mb and accounting for 34.34% of SSRs.

Dinucleotide SSRs were found to have the highest densities, ranging from 956.40 to 10,152.94 bp/Mb in *R. ferrugineus*, *S. oryzae*, *H. hampei*, *I. nitidus*, *L. oregonensis*, and *L. bonariensis* (Figure 3C). Trinucleotide SSRs had the highest densities (198.78–561.66 bp/Mb) in *D. ponderosae* and *P. strobi*, whereas tetranucleotide SSRs had the highest density (91.25 bp/Mb) in *E. kamerunicus* (Figure 3C). Across the investigated genomes, hexanucleotide SSRs were the least abundant at frequencies below 1.93 SSRs/Mb, except in *L. oregonensis*, for which pentanucleotide SSRs were identified to be the least frequent (1.08 SSRs/Mb).

Next, GC content was investigated for the various types of SSRs (Figure 3D). The highest GC content was observed for hexanucleotide SSRs, which had values of 19.48–54.34%, except in *P. strobi*, for which genome mononucleotide SSRs exhibited the highest GC content at 43.26%. Meanwhile, the lowest levels of GC content were identified for dinucleotide SSRs in *S. oryzae*, *R. ferrugineus*, and *L. oregonensis*, at values of only 0.55–4.41%; for mononucleotide SSRs in *H. hampei*, *E. kamerunicus*, *L. bonariensis*, and *D. ponderosae*, at 0.01–12.24%; and for trinucleotide SSRs in *P. strobi*, at 8.82%.

### 3.3. Diversity of Microsatellite Motifs in Beetle Genomes

The microsatellites in the weevil genome assemblies examined here were found to be relatively AT-rich. To gain insight into this characteristic, we further analyzed the motif composition of SSRs. Motif abundance was found to vary across the draft genomes. More specifically, the investigated assemblies were identical in the degenerated number of repeat motifs for mono- to trinucleotide SSRs, at 2, 4, and 10 motifs respectively, but differed in the number of tetranucleotide, pentanucleotide, and hexanucleotide repeat motifs.

**Figure 4:**
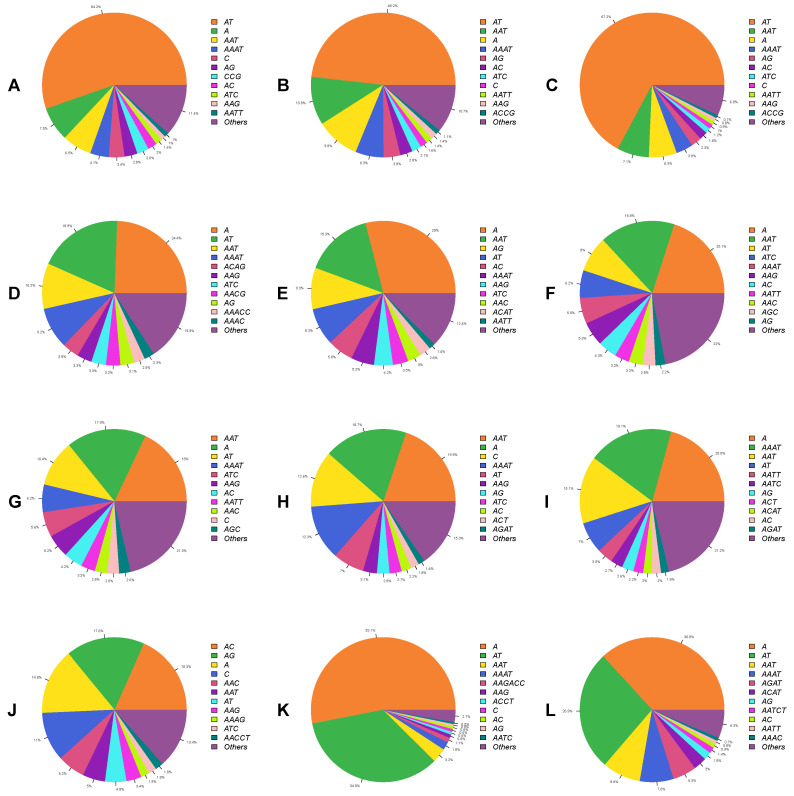
Percentages of top SSR motif types in the weevil draft genomes. Percentages were calculated by dividing the number of SSRs of each motif type by the total number of SSRs in each assembly. (**A**–**L**) represent the weevil draft genomes *R. ferrugineus* (F), *R. ferrugineus* (M), *R. ferrugineus* (L), *S. oryzae*, *H. hampei*, *D. ponderosae* (F), *D. ponderosae* (M), *P. strobi*, *E. kamerunicus*, *I. nitidus*, *L. oregonensis*, and *L. bonariensis*.

Among mononucleotide repeats, the predominate motif was (A)_n_, with total counts of 4385, 4951, 4228, 20603, 3798, 1305, 1215, 28851, 913, 7138, 283468, and 57712 SSRs in *R. ferrugineus* (F), *R. ferrugineus* (M), *R. ferrugineus* (L), *H. hampei*, *D. ponderosae* (F), *D. ponderosae* (M), *P. strobi*, *E. kamerunicus*, *I. nitidus*, *L. oregonensis*, and *L. bonariensis* respectively. This type accounted for 6.29–53.07% of all mononucleotide SSRs in the draft genomes (Figure 4). The frequency of the (A)_n_ motif ranged from 3.39–219.19% SSRs/Mb, with the highest frequency observed in *L. oregonensis* and the lowest in *E. kamerunicus*. The (C)_n_ motif type was far less abundant, accounting for just 0.01–12.62% of all mononucleotide SSRs in the twelve draft genomes.

Among dinucleotide SSRs, the most prominent type in ten draft genomes was the (AT)_n_ motif, with frequencies ranging from 1.14 to 142.38 SSRs/Mb; the exceptions were *H. hampei* and *I. nitidus*, in which this motif comprised about 6.98–67.17% of dinucleotide SSRs (Figure 4). In *H. hampei*, the most frequent dinucleotide motif was the (AG)_n_ repeat at 7.47 SSRs/Mb, accounting for 9.28% of all SSRs in that assembly. Meanwhile, in *I. nitidus*, the most prevalent dinucleotide motif was (AC)_n_ with frequency 25.72 SSRs/Mb; this motif accounted for 18.35% of all dinucleotide SSRs in that genome. Notably, the (AG)_n_ repeat was almost equally frequent in *I. nitidus* (24.67 SSRs/Mb). In all weevil assemblies, the least frequent dinucleotide SSR was the (CG)_n_ motif.

For the trinucleotide repeat type, the (AAT)_n_ repeat was the most frequent motif in eleven draft genomes, with frequencies ranging from 3.36 to 15.20 SSRs/Mb; these repeats accounted for 3.24–19.92% of all trinucleotide SSRs Figure 4). The exception was *I. nitidus*, in which the (AAC)_n_ repeat was the most frequent trinucleotide motif, followed by the (AAT)_n_ motif; these had frequencies of below 9 SSRs/Mb, and together accounted for 11.24% of all trinucleotide SSRs in that species.

Among tetranucleotide repeats, (AAAT)_n_ was the most abundant in eleven assemblies with frequencies ranging from 1.69 to 10.65 SSRs/Mb and accounting for 1.81–19.06% of all tetranucleotide SSRs. The exception was again *I. nitidus* (Figure 4), in which the most frequent tetranucleotide motif was (AAAG)_n_, with frequency 2.66 SSRs/Mb and comprising about 1.89% of all tetranucleotide SSRs in that draft genome.

For pentanucleotide repeats, the most abundant motifs varied among species. (AAACC)_n_ was the most abundant in the *S. oryzae*, with frequency of 2.68 SSRs/Mb and comprising about 2.45% of pentanucleotide SSRs in this draft genome. The (AATAT)_n_ motif was the most frequent in the *R. ferrugineus* adult female, *R. ferrugineus* adult male and *R. ferrugineus* larva with frequencies of 0.19, 0.33, and 0.43 SSRs/Mb, respectively. Meanwhile, (AAATC)_n_ and (AAATT)_n_ motifs had similar frequencies of approximately 0.15 SSRs/Mb in the *D. ponderosae* adult female and male assemblies, accounting for 1.92% of all pentanucleotide SSRs. (AACCT)_n_ repeats were the predominant pentanucleotide motif in *H. hampei* and *I. nitidus*, with frequencies below 3 SSRs/Mb. (ACGAG)_n_ and (AATCT)_n_ motif types were more abundant in the *L. oregonensis*, and *L. bonariensis*, with respective frequencies of 1.22 and 1.90 SSRs/Mb. Finally, *P. strobi* and *E. kamerunicus* were found to share their most frequent pentanucleotide motif, (AAATC)_n_, with a frequency below 0.6 SSRs/Mb.

Hexanucleotide motifs occurred at a far lower frequency in the examined weevil genomes than did other microsatellite repeat types. The (AAACCC)_n_ motif was the most abundant hexanucleotide in the *R. ferrugineus* adult female and *R. ferrugineus* larva draft genomes, with frequencies of less than 0.07 SSRs/Mb, while the (ACATAT)n repeat was the most frequent in the *R. ferrugineus* adult male, with the frequency of 0.03 SSRs/Mb. The (AAATTC)_n_ motif was the most frequent type in *D. ponderosae*, *P. strobi*, *E. kamerunicus*, and *L. bonariensis*, with frequencies below 0.4 SSRs/Mb. Meanwhile, (AAGAGG)_n_, (ACACAT)_n_, (AAAGAG)_n_, and (AAGACC)_n_ motifs were the most abundant hexanucleotide repeats in *S. oryzae*, *H. hampei*, *I. nitidus*, and *L. oregonensis*, respectively.

### 3.4. Microsatellite Distribution and Motif Diversity According to Genomic Region

**Figure 5:**
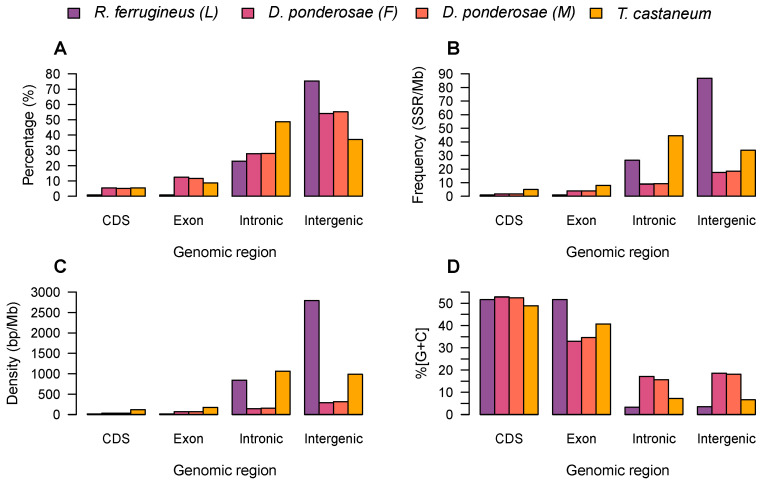
Comparison of SSR percentage, frequency, density, and GC content among different genomic regions in four beetle draft genomes. (**A**–**D**) represent the percentage, frequency, density, and GC content of SSRs, respectively.

The distribution of SSRs across different genomic regions was investigated in four draft genomes representing three species (*R. ferrugineus* larva, female and male *D. ponderosae*, and *T. castaneum*) as described in the Methods. Specifically, microsatellite analysis was executed to examine the distribution of SSRs in exons, CDSs, and intronic and intergenic regions. The results revealed most mono- to hexanucleotide SSRs to have region-associated differences in terms of their relative abundance, density, and percentage, and those differences to vary between species; however, as expected, results in the female and male *D. ponderosae* were substantially similar. Overall, lower relative frequencies and densities of SSRs were observed in coding and noncoding regions than in intronic and intergenic regions (Figure 5). Microsatellites were most commonly identified in intergenic regions, followed in order by intronic regions, exons, and CDSs, with one exception: SSRs were found to be abundant in the intronic regions of *T. castaneum* (Figure 5B). In CDSs of the four assemblies, SSR frequency ranged from 0.95 to 4.97 SSRs/Mb; overall, coding regions contained 0.83–5.54% of SSRs. In exons, SSR frequency ranged from 0.95 to 3.90 SSRs/Mb except in *T. castaneum*, which had a frequency 8.03 SSRs/Mb; collectively, exonic regions accounted for 0.83–12.44% of SSRs in the four samples. In intronic regions of *R. ferrugineus* larva, female and male *D. ponderosae*, and *T. castaneum*, respectively, the observed SSR frequencies were 26.47, 8.99, 9.36, and 44.50 SSRs/Mb; in total, introns accounted for 22.98–27.98% of SSRs except in *T. castaneum*, where they comprised 48.71%. Finally, intergenic regions exhibited respective frequencies of 26.47, 8.99, 9.36, and 44.50 SSRs/Mb, and accounted for 37.05–75.37% of SSRs in the four assemblies. Overall, microsatellite densities were higher in noncoding regions than in coding regions: intronic regions had densities of 146.19–1063.02 bp/Mb, and intergenic regions of 289.79–2791.55 bp/Mb, while CDSs had densities of 15.58–119.29 bp/Mb and exons of 15.58–177.18 bp/Mb (Figure 5C).

Next, the GC content of microsatellites was examined according to genomic region (Figure 5C). Across the four assemblies, GC contents were mostly identical in coding regions (CDSs and exons), but were found to vary in noncoding regions (intronic and intergenic regions). The highest GC contents were observed for SSRs located in CDSs (48.88–52.85%), followed by those in exons (32.98–51.66%), whereas intronic regions had GC contents of 3.31–17.16% and intergenic regions of 3.51–18.61%.

**Figure 6:**
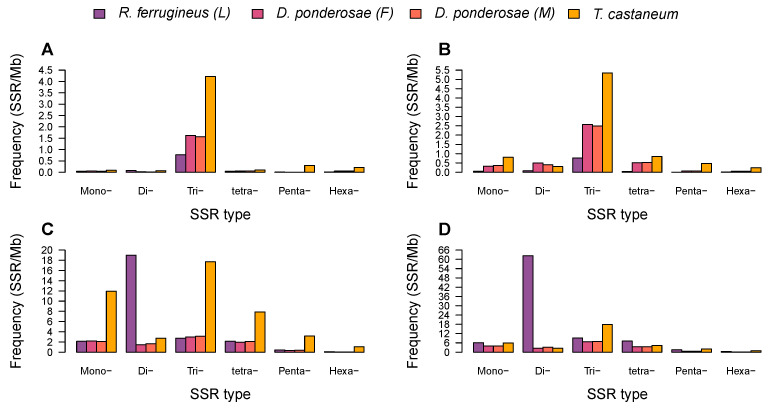
Relative frequency of mono- to hexanucleotide SSRs in different genomic regions of four draft beetle genomes. (**A**–**D**) represent CDSs, exons, intronic regions, and intergenic regions, respectively.

Among CDSs and exons, trinucleotide SSRs were the most abundant type (0.77–5.35 SSRs/Mb) in all four genomes, while pentanucleotide SSRs were consistently the least frequent in the three curculionid assemblies (Figure 6A,B). For the tenebrionid *T. castaneum*, di- and hexanucleotide SSRs were the least abundant types in CDSs (0.07 SSRs/Mb) and exons (0.24 SSRs/Mb), respectively. In intronic and intergenic regions, trinucleotide SSRs were the most abundant type in *D. ponderosae* and *T. castaneum*, with frequencies of 17.94–2.99 SSRs/Mb, whereas dinucleotide SSRs were the most abundant type in *R. ferrugineus* (Figure 6C,D). Pentanucleotide SSRs were rare in intronic and intergenic regions, and hexanucleotide SSRs were the least abundant, with frequencies below 1.08 SSRs/Mb for all four genomes (Figure 6C,D).

**Figure 7:**
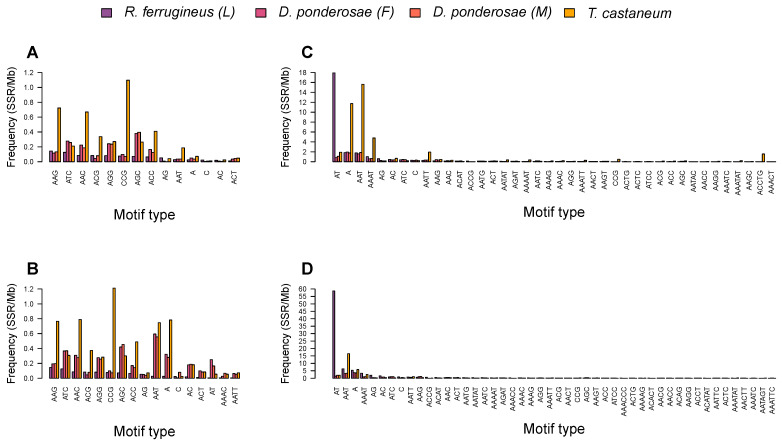
The most frequent SSR motif types in different genomic regions of four draft beetle genomes. (**A**–**D**) represent CDSs, exons, intronic regions, and intergenic regions, respectively.

Among the three beetle species examined here, motif types were found to vary quite obviously in different genomic regions (Figure 7). In coding regions of *R. ferrugineus* and *T. castaneum*, the predominant motifs were (AAG)_n_ and (CCG)_n_, respectively, accounting for 15–22% of CDS and exonic SSRs (Figure 7A,B). Meanwhile, (AGC)_n_ and (AAT)_n_ respectively comprised the most abundant trinucleotide repeats in the CDSs and exonic regions of *D. ponderosae*. In noncoding regions of the *R. ferrugineus* genome, the (AT)_n_ motif was the most abundant repeat, representing ∼67% of intronic and intergenic SSRs (Figure 7C,D). Meanwhile, intronic and intergenic regions of the *T. castaneum* assembly had (AAT)_n_ as the most common repeat, with frequencies of approximately 16 SSRs/Mb. In *D. ponderosae* assemblies, (A)_n_ and (AAT)_n_ were the most abundant motifs in intronic regions and intergenic regions, with frequencies below 4 SSRs/Mb.

## 4. Discussion

The development of next-generation sequencing has allowed for the generation of a massive number of sequenced draft genomes, including those of non-model species. The availability of draft genomic sequences from Curculionidae weevils allowed us to investigate the distributions of microsatellites in members of this family. As far as we know, this is the first comprehensive report on the identification and analysis of SSRs 1–6 bp long in the entire draft genomes of nine curculionid beetles. We used computational techniques to search for microsatellites and compare the relative frequency, relative density, and GC content of SSRs in these beetles. Consistent search parameters were utilized so as to carry out the same analysis in each investigated draft genome. BUSCO results suggest these draft genomes are mostly comparable. Moreover, BUSCO indicated that our female RPW assembly is more complete than male RPW (GCA_012979105.1) [27] both in terms of complete single genes (92.2% versus 52.9%, respectively) and of missing genes (3.0% versus 15.13%, respectively). SSR repeat content differs between species, which might be a general phenomenon across taxa [33]. Previous studies reported SSRs to comprise 3% of the human genome [34], 0.04–0.44% of plant and fungal genomes [35,36,37], and 0.44–0.88% of primate genomes [22,38]. Here, our results showed that identified SSRs differ with the degree of coverage and comprise 0.02–1.44% of the draft genomes for these nine weevil species. Assemblies representing the same species exhibited similar proportions of SSRs, as seen in female and male *D. ponderosae*, whereas values differed between species. The observed variance in microsatellite proportion could result from differences in computational approaches utilized for SSR detection, incompleteness of genome assemblies, or actual variation in SSR content among these weevils [39]. Moreover, variation might even arise between closely related species [40,41].

Our findings suggest that in weevils, the number of SSRs is significantly positively correlated with genome size; this is inconsistent with the results reported in [35,42]. Nonetheless, a study reported that the number of SSRs was significantly associated with genome size in 136 insects [43], which agrees with our results. However, it is necessary to sequence more genomes of beetles from the Curculionidae family to solidify this conclusion. In this work, frequency and density of SSRs were not significantly correlated with genome size.

In all of the weevil species examined here, the six types of SSRs were not evenly distributed; rather, mono- to trinucleotide SSRs were the most prevalent. This finding is consistent with previous reports that mono- to trinucleotide SSR repeats are more frequent in 23 mosquito species [44] and six plant species [45]. Meanwhile, tetra- to hexanucleotide SSRs were the least frequent types in these draft genomes, an observation similar to what has been found in Palmae genomes [35] and *Gossypium* species [46]. More specifically, we observed dinucleotide SSRs to be the most frequent repeat type in *R. ferrugineus* and *I. nitidus*, consistent with dicotyledons [47] and *Drosophila* [14]. Mononucleotide SSRs were the dominant type in *S. oryzae*, *P. strobi*, *L. oregonensis*, and *L. bonariensis*, which is consistent with prior findings for *Batocera horsfieldi* [48] and eukaryotic genomes [39,49]. Finally, trinucleotide SSRs were the most abundant type in *H. hampei*, which is consistent with eukaryotes [50]. The higher abundance of SSRs with shorter motif lengths (mono-, di-, and trinucleotides) could be the result of a higher frequency of replication slippage over shorter repeat monomers. Additionally, repeat motifs may differ in the stability of secondary structures they form, which might also impact the evolutionary dynamics of their abundance and distribution [51]. However, no such analysis has been performed in weevils and the relative contributions of selection and the molecular mechanisms affecting the abundance of SSRs (e.g., slippage, rolling circle amplification, crossing over, gene conversion) is poorly understood in general [52].

We also observed SSR motifs within each microsatellite type to vary in abundance across the examined draft genomes. Among mononucleotide repeats, the most frequent motif was (A/T)_n_, occupying about 6.29–53.07% of mononucleotide SSRs in these genomes, similar to the trend previously reported across 100 insect species [43]. Of dinucleotide SSRs, the most abundant motifs were (AT)_n_ and (AG)_n_, similar to palms [35], several insect species [43], and garden asparagus [53]. Regarding trinucleotide motifs, (AAT)_n_ was the dominant motif in most weevil draft genomes, which is consistent with both mammals [22,49] and plants [35,54]. Of tetra-, penta-, and hexanucleotide SSRs, (AAAT)_n_, (AAAG)_n_, (AAACC)_n_, (AAATC)_n_, (AAATT)_n_, (AATAT)_n_, (AATCT)_n_, (ACATAT)_n_, and (AAATTC)_n_ were the more frequent motifs. Overall, these findings are consistent with previous reports suggesting that AT-rich SSR motifs predominate [43,48]. The abundance of AT-rich SSRs might also reflect the overall base composition of insect genomes, which are often AT-rich themselves [43].

Strong evidence exists that the microsatellites are nonrandomly distributed across protein-coding regions, untranslated regions, and introns, and that they may play roles in gene expression and regulation [55,56]. Moreover, SSRs may play different functional roles in different genomic regions. We further investigated the distribution of SSRs in different genomic regions for four beetles from three species representing two beetle families (Curculionidae and Tenebrionidae). We found SSR abundance to differ among genomic regions in these genomes; moreover, the same genomic regions in different species showed notable similarity in SSR distribution, consistent with previous studies in mammals and plants [49,57]. SSRs were found to occur less frequently in coding regions than in noncoding regions, which aligns with previous reports [58,59]. Specifically, SSRs were greatly abundant in intergenic and intronic regions, less common in exons, and least abundant in CDSs. These results may suggest that SSRs in coding regions are subject to negative/purifying selection pressure [23].

Within CDSs and exons, trinucleotide SSRs were the most abundant repeat type, which echoes results from prior studies in mammals and plants [23,49]. The predominance of trinucleotide SSRs in coding regions may be due to frameshift mutations eliminating non-trimeric SSRs [60]. Inconsistent with previous reports in mosquitos, primates, mammals, and plants [35,44,49,58], we observed intronic and intergenic regions to feature trinucleotide SSRs as the most abundant repeat type in *D. ponderosae* and *T. castaneum*, but dinucleotide SSRs in *R. ferrugineus*.

Notably, SSRs exhibit bias toward a few specific nucleotide motifs according to the genomic region they occur in. In coding regions of the *R. ferrugineus* and *T. castaneum* genomes, (AAG)_n_ and (CCG)_n_ repeats predominated; meanwhile, there was a noticeable excess of the (AGC)_n_ motif in the CDSs and exonic regions of *D. ponderosae*, similar to observations in *Drosophila* [14]. Consistent with previous reports [35,58,59], AT-rich motifs such as (AT)_n_, (AAT)_n_, and (A)_n_ were the most abundant in the intronic and intergenic regions of the examined beetle genomes, which can be interpreted as confirming high AT content in the majority of the analyzed SSRs.

To evaluate the effects of nucleotide composition on SSR abundance, we examined GC content in relation to SSR type in the different genomic compartments of all nine weevil species. The results showed average GC content values (0.01–54.34%) to be much lower than AT content values, and moreover that the distribution of GC content was uneven; this is consistent with previous reports [35,49,58,59]. The greatest GC content values were mostly detected among hexanucleotide SSRs and the least for mono- and dinucleotide SSRs. In terms of genomic regions, CDSs demonstrated the most GC content, followed by exonic regions, then intergenic regions, and lastly intronic regions. These results suggest that high GC content is more frequently distributed in coding regions, consistent with results reported in [58]. The bias for high GC in coding regions has been suggested to increase the bendability of the double helix [61] and in turn contribute to maintain the higher transcriptional activity in these regions [62].

All told, this study performed the first comprehensive large-scale analysis of microsatellites in draft genomes of nine crop pests of the Curculionidae family, with a focus on common features of SSRs including their abundance patterns and variation characteristics. The findings of this work provide useful insights into the diversity and distributions of SSRs in these weevil species. The SSR number in these draft genomes was significantly correlated with genome size and but not significantly correlated with GC content. Mono- to trinucleotide SSRs were dominant in all examined species, but the occurrence, percentage, and density of each type of SSR varied between species. Overall, most SSRs were distributed in intronic and intergenic regions; within coding regions, trinucleotide SSRs predominated. Genomic microsatellite markers are widely used in population genetics and evolutionary studies because they are reliable, highly polymorphic, and easy to amplify [63]. Further refining our understanding of the characteristics of SSRs in weevil genomes will serve as a foundation for genetic research and the selection of SSR molecular markers in these beetles.

## Figures and Tables

**Table 1:** Overview of draft genomes for the nine weevil species.

Insect Name	Common Name	Genome Size (Mb)	Number of SSRs	Frequency (SSR/Mb)	Density (bp/Mb)	SSRs Content (%)	Reference
*R. ferrugineus* (F)	Female red palm weevil	1121.36	57,175	50.99	1320.92	0.13	This study
*R. ferrugineus* (M)	Male red palm weevil	782.10	50,723	64.86	1445.93	0.14	[27]
*R. ferrugineus* (L)	Red palm weevil larva	589.40	67,261	114.11	3649.45	0.36	[28]
*S. oryzae*	Rice weevil	770.57	84,391	109.52	3287.11	0.33	[29]
*H. hampei*	Coffee berry borer	162.57	13,092	80.53	3260.24	0.33	[30]
*D. ponderosae* (F)	Female mountain pine beetle	223.74	6505	29.07	481.68	0.05	Unpublished
*D. ponderosae* (M)	Male mountain pine beetle	224.79	6803	30.26	511.44	0.05	Unpublished
*P. strobi*	White pine weevil	2025.02	154,511	76.30	1516.87	0.15	Unpublished
*E. kamerunicus*	African oil palm weevil	269.64	4397	16.31	249.98	0.02	[31]
*I. nitidus*	Qinghai spruce bark beetle	345.00	48372	140.21	3127.27	0.31	Unpublished
*L. oregonensis*	Carrot weevil	1293.28	534,123	412.99	14,406.26	1.44	Unpublished
*L. bonariensis*	Argentine stem weevil	1112.44	156,716	140.88	3976.45	0.40	[32]

**Table 2:** Number, length, frequency, and density of SSRs by type (mono- to hexanucleotide repeats) in the investigated weevil genomes.

Repeat Type	Parameter	*R. ferrugineus* (F)	*R. ferrugineus* (M)	*R. ferrugineus* (L)	*S. oryzae*	*H. hampei*	*D. ponderosae* (F)	*D. ponderosae* (M)	*P. strobi*	*E. kamerunicus*	*I. nitidus*	*L. oregonensis*	*L. bonariensis*
Mono-	Number of SSRs	6393	5748	4906	22,484	3924	1415	1395	48,345	939	12,459	286,859	57,721
	Total length (bp)	98,929	79,577	69,520	429,331	70,509	18,625	18,802	725,181	11,752	178,828	4,316,826	809,526
	Average length (bp)	15.47	13.84	14.17	19.09	17.97	13.16	13.48	15.00	12.52	14.35	15.05	14.02
	Frequency (SSR/Mb)	5.70	7.35	8.32	29.18	24.14	6.32	6.21	23.87	3.48	36.11	221.81	51.89
	Density (bp/Mb)	88.22	101.75	117.95	557.16	433.71	83.24	83.64	358.11	43.58	518.33	3337.89	727.70
Di-	Number of SSRs	34,681	27,781	47,993	196,96	3077	944	1128	18,612	517	19,728	190,064	46,373
	Total length (bp)	1,084,748	748,002	1,823,936	1,157,184	335,416	15,834	19,970	360,806	7412	493,798	13,130,600	2,463,404
	Average length (bp)	31.28	26.92	38.00	58.75	109.00	16.77	17.70	19.39	14.34	25.03	69.09	53.12
	Frequency (SSR/Mb)	30.93	35.52	81.43	25.56	18.93	4.22	5.02	9.19	1.92	57.18	146.96	41.69
	Density (bp/Mb)	967.35	956.40	3094.55	1501.73	2063.20	70.77	88.84	178.17	27.49	1431.28	10,152.94	2214.42
Tri-	Number of SSRs	8517	8897	7407	15,786	4028	2558	2663	45,395	1050	10,357	23,752	16,301
	Total length (bp)	142,764	149,838	126,207	284,631	79,143	44,475	46,941	1,137,378	15,882	196,965	457,914	304,446
	Average length (bp)	16.76	16.84	17.04	18.03	19.64821	17.39	17.63	25.06	15.13	19.02	19.28	18.68
	Frequency (SSR/Mb)	7.60	11.38	12.57	20.49	24.78	11.43	11.85	22.42	3.89	30.02	18.37	14.65
	Density (bp/Mb)	127.31	191.58	214.13	369.38	486.82	198.78	208.82	561.66	58.90	570.90	354.07	273.67
Tetra-	Number of SSRs	5368	6700	5515	19,698	1627	1310	1323	32,698	1510	3915	21,085	29,736
	Total length (bp)	94,688	116,572	97,284	422,340	30,380	22,688	22,752	621,160	24,604	95,912	406,368	654,160
	Average length (bp)	17.64	17.40	17.64	21.44	18.67	17.32	17.20	18.99	16.29	24.50	19.27	21.99
	Frequency (SSR/Mb)	4.79	8.57	9.36	25.56	10.01	5.86	5.89	16.15	5.60	11.35	16.30	26.73
	Density (bp/Mb)	84.44	149.05	165.06	548.09	186.87	101.40	101.21	306.74	91.25	278.00	314.21	588.04
Penta-	Number of SSRs	1318	1324	1151	5375	382	230	239	6630	348	1289	5786	5784
	Total length (bp)	30,555	28,995	25,330	135,845	13,175	4900	5145	147,940	6960	70,845	136,620	158,590
	Average length (bp)	23.18	21.90	22.01	25.27	34.49	21.30	21.52	22.31	20.00	54.96	23.61	27.42
	Frequency (SSR/Mb)	1.18	1.69	1.95	6.98	2.35	1.03	1.06	3.27	1.29	3.74	4.47	5.20
	Density (bp/Mb)	27.25	37.07	42.98	176.29	81.04	21.90	22.89	73.06	25.81	205.35	105.64	142.56
Hexa-	Number of SSRs	898	273	289	1352	54	48	55	2831	33	624	6577	801
	Total length (bp)	29,538	7872	8718	103,614	1398	1248	1356	79,230	792	42,576	183,012	33,432
	Average length (bp)	32.89	28.84	30.17	76.64	25.89	26.00	24.65	27.99	24.00	68.23	27.83	41.74
	Frequency (SSR/Mb)	0.80	0.35	0.49	1.75	0.33	0.21	0.24	1.40	0.12	1.81	5.09	0.72
	Density (bp/Mb)	26.34	10.07	14.79	134.47	8.60	5.58	6.03	39.13	2.94	123.41	141.51	30.05

## Data Availability

Data generated and analyzed during this study are included in the published article, its Supplementary Files, and publicly available repositories. Raw reads from genome sequencing of the female *R. ferrugineus* have been deposited at NCBI Sequence Read Archive (SRA) under the BioProject accessions PRJNA848948. Draft genome of the female *R. ferrugineus* weevil can be found at https://doi.org/10.5281/zenodo.6878576 (accessed on 21 July 2022).

## Supplementary Materials

The supporting information can be downloaded at: https://www.mdpi.com/article/10.3390/ijms23179847/s1.
